# Identification of a periodontal pathogen and bihormonal cells in pancreatic islets of humans and a mouse model of periodontitis

**DOI:** 10.1038/s41598-020-65828-x

**Published:** 2020-06-19

**Authors:** Vladimir Ilievski, Peter T. Toth, Klara Valyi-Nagy, Tibor Valyi-Nagy, Stefan J. Green, Rosann S. Marattil, Haider W. Aljewari, Barton Wicksteed, Neil M. O’Brien-Simpson, Eric C. Reynolds, Brian T. Layden, Terry G. Unterman, Keiko Watanabe

**Affiliations:** 1 Department of Periodontics, College of Dentistry, University of Illinois at Chicago, Chicago, Illinois USA; 2 Department of Pharmacology, College of Medicine, University of Illinois at Chicago, Chicago, Illinois USA; 3 Fluorescence Imaging Core Facility, University of Illinois at Chicago, Chicago, Illinois USA; 4 Department of Pathology, College of Medicine, University of Illinois at Chicago, Chicago, Illinois USA; 5 Department of Biological Sciences, University of Illinois at Chicago, Chicago, Illinois USA; 6 DNA Core Facility, University of Illinois at Chicago, Chicago, Illinois USA; 7 Undergraduate Program, University of Illinois at Chicago, Chicago, Illinois USA; 8 Post-Gradulate Program in Periodontics, College of Dentistry, University of Illinois at Chicago, Chicago, Illinois USA; 9 Division of Endocrinology, Diabetes & Metabolism, College of Medicine, University of Illinois at Chicago, Chicago, Illinois USA; 10 Melbourne Dental School, University of Melbourne, Melbourne, Victoria Australia; 11 Jesse Brown VA Medical Center, Chicago, Illinois USA

**Keywords:** Endocrinology, Pathogenesis

## Abstract

Results from epidemiological and prospective studies indicate a close association between periodontitis and diabetes. However the mechanisms by which periodontal pathogens influence the development of prediabetes/diabetes are not clear. We previously reported that oral administration of a periodontal pathogen, *Porphyromonas gingivalis* (Pg) to WT mice results in insulin resistance, hyperinsulinemia, and glucose intolerance and that Pg translocates to the pancreas. In the current study, we determined the specific localization of Pg in relation to mouse and human pancreatic α- and β-cells using 3-D confocal and immunofluorescence microscopy and orthogonal analyses. Pg/gingipain is intra- or peri-nuclearly localized primarily in β-cells in experimental mice and also in human post-mortem pancreatic samples. We also identified bihormonal cells in experimental mice as well as human pancreatic samples. A low percentage of bihormonal cells has intracellular Pg in both humans and experimental mice. Our data show that the number of Pg translocated to the pancreas correlates with the number of bihormonal cells in both mice and humans. Our findings suggest that Pg/gingipain translocates to pancreas, particularly β-cells in both humans and mice, and this is strongly associated with emergence of bihormonal cells.

## Introduction

Periodontitis is a disease characterized by destruction of gingiva and tooth-supporting bone by a host immunological response triggered by periodontal pathogens. Thus, the primary etiological factor of periodontitis is bacteria. Interestingly, certain periodontal pathogens are detected in different organs such as the liver^1^, aorta^2^, arteries^3,4^ and the brain^5^, but the effects of periodontal pathogens in these tissues and organs are poorly understood.

One of the major periodontal pathogens, *Porphyromonas gingivalis* (Pg), is a non-motile gram-negative obligate anaerobic bacteria that possesses virulence factors including cysteine proteases referred to as gingipains (arginine specific gingipain, RgpA/B and lysine specific gingipain, Kgp) which are associated with the outer cell membrane and membrane vesicles^6^. It has been reported that a heterodimer of gingipains, HRgp, has the ability to enter the nucleus of epithelial cells *in vitro*^7^, but its function in the nucleus is not known. We have recently shown that Pg orally administered to mice is translocated to the brain and is localized peri- and intra-nuclearly in neurons, astrocytes and microglial cells in the hippocampus and this is associated with the development of senile plaques^5^. This suggests that Pg/gingipain is able to invade cells in distant organs and may result in a pathological conditions. In regards to a relationship between periodontal pathogens and the human pancreas, the presence of a periodontal pathogen, *Fusobacterium* species was reported in human pancreatic ductal adenocarcinomas and cyctic fluid from Intraductal papillary mucinous neoplasm^8,9^. Although the presence of Pg in the pancreas has not been investigated, increased antibody to Pg has been detected in the plasma of subjects with pancreatic cancer^10^.

We have recently determined that mice orally administered Pg develop insulin resistance and hyperinsulinemia while maintaining normal glucose levels indicating a prediabetic condition^11^ and that Pg translocates to the pancreas^12^. These results suggest that Pg may influence β-cell function. To gain understanding of how Pg interacts with islet cells, we set out to determine the specific localization of Pg in α- and β-cells in mouse pancreatic islets and human pancreatic islet cells. In this process we quantitated the relative number of α- and β-cells containing Pg and the emergence of bihormonal cells which express both insulin and glucagon in response to translocated Pg.

The emergence of bihormonal cells in animal models has been reported following near complete destruction of β-cells (99% ablation) by chemical agent^13^ or by forced expression/deletion of β- or α-cell specific transcription factors^14–17^ using conditional knockout and/or lineage tracing mice. Re-differentiation of α-cells from de-differentiated β-cells^18^ also represents another means of developing intermediate/bihormonal cells. Beta- to α-cell conversion has also been reported as a result of DNMT1 deletion^19^. Taken together, these studies show plasticity of pancreatic islet cells under defined conditions. Most recently, emergence of bihormonal cells was observed in a mouse model of experimental autoimmune diabetes^20^. In contrast to animal studies, quantitative data on human pancreatic bihormonal cells are scarce^21,22^. A recent study using human pancreatic samples obtained following pancreatoduodenectomy reported the higher percentage of bihormonal cells in an insulin resistant group compared with an insulin sensitive group, suggesting a possible adaptive response to insulin resistance^23^.

Here we show that orally applied Pg in mice translocates to and resides in intra- and peri-nuclear locations primarily in islet β-cells. The emergence of bihormonal cells was strongly associated with the presence of Pg/gingipain in pancreatic islets of these animals as well as in human post-mortem pancreatic samples. These observations support the novel concept that oral bacteria causing periodontal infection can translocate to pancreatic islets where they may impact islet pathophysiology and the development of bihormonal cells.

## Results

### Pg/gingipain translocates to nuclear- and peri-nuclear regions of β-cells but not to α-cells in animals administered Pg

**Figure 1:**
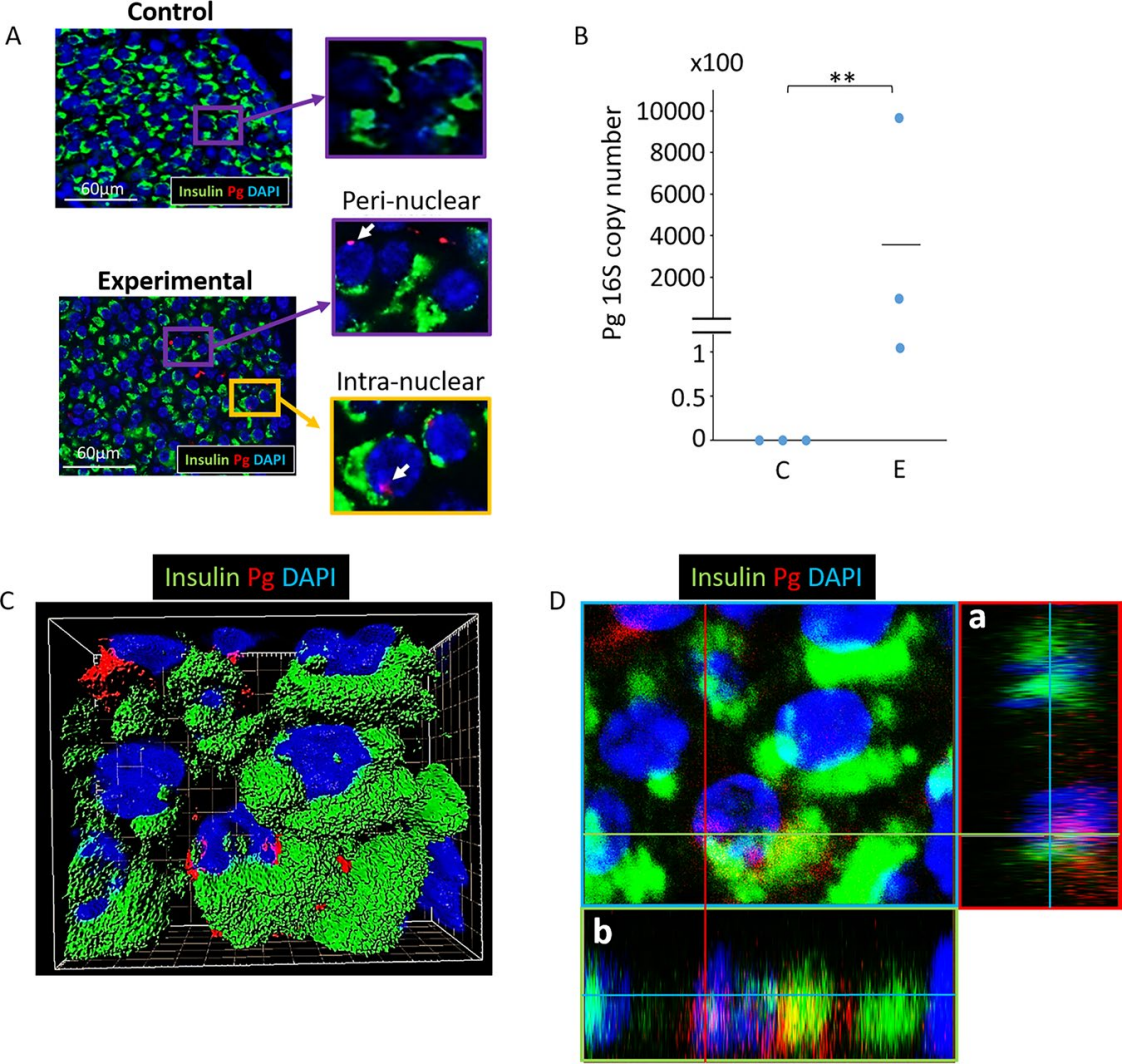
Pg/gingipain translocates to the pancreas and is present in β-cells. (**A**) Representative result using IF microscopy showing Pg/gingipain in pancreatic islets in experimental but not control animals. White arrows point to Pg/gingipain. Scale bar represents 60μm. N = 9 mice/experimental group and N = 10 mice/control group. (**B**) Results from qPCR for Pg 16 S rRNA genes performed on formalin fixed paraffin embedded (FFPE) sections (5/animal) show that Pg is present in islets from experimental animals but not controls. N = 3 mice/group. The difference between groups is statistically significant (p** < 0.01). (**C**) Representative 3-D image from confocal microscopy of mouse pancreatic islet showing that Pg is localized in β-cells. (**D**) Orthogonal analysis of image confirming intra-nuclear localization of Pg in β-cells. a & b are orthogonal projections showing the side views of the main image cut through along vertical (red) and horizontal (green) lines. Green: insulin, Red: Pg, Blue: nuclei.

Following oral application of Pg 3 times per week for 22 weeks to simulate chronic periodontitis, the presence of Pg/gingipain was determined. Pg/gingipain was identified in pancreata of all mice that were administered Pg (N = 9) but none in control mice treated with vehicle alone (N = 10) by immunofluorescence (IF) microscopy and qPCR (N = 3/group) (Fig. 1A,B). 3-D confocal microscopy and orthogonal analyses revealed nuclear- or peri-nuclear localization of Pg/gingipain in β-cells (Fig. 1C,D, respectively).

**Figure 2:**
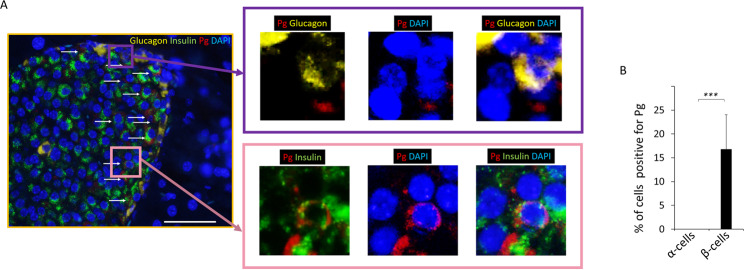
Pg is localized in β- but not in α-cells in experimental animals. (**A**) Representative image from IF microscopy and higher magnification of insets showing Pg, glucagon, and nuclear stain (top panels) and Pg, insulin and nuclear staining (bottom panels). Yellow: glucagon, Green: insulin, Red: Pg, Blue: nuclei. White arrows point to Pg in low magnification image. Scale bar represents 50μm. For separate channel images of 40×, see Fig. S1. (**B**) Results from counting cells positive for Pg/gingipain. The total number of insulin+ cells and insulin+/Pg+ cells were counted per islet and the percentage of insulin+ cells that were positive for Pg were calculated (5 islets /mouse, N = 5 mice/group). Data show mean + S.D. There were no glucagon+ cells containing Pg. The difference in the percentage of α- vs. β-cells containing Pg is statistically significant (***p < 0.001).

Interestingly, Pg/gingipain was localized intracellularly in β-cells but not in α-cells that were identified based on insulin and glucagon staining, respectively (p < 0.001) (Fig. 2A,B). Again, no Pg/gingipain was detected in pancreatic samples from control animals.

**Figure 3:**
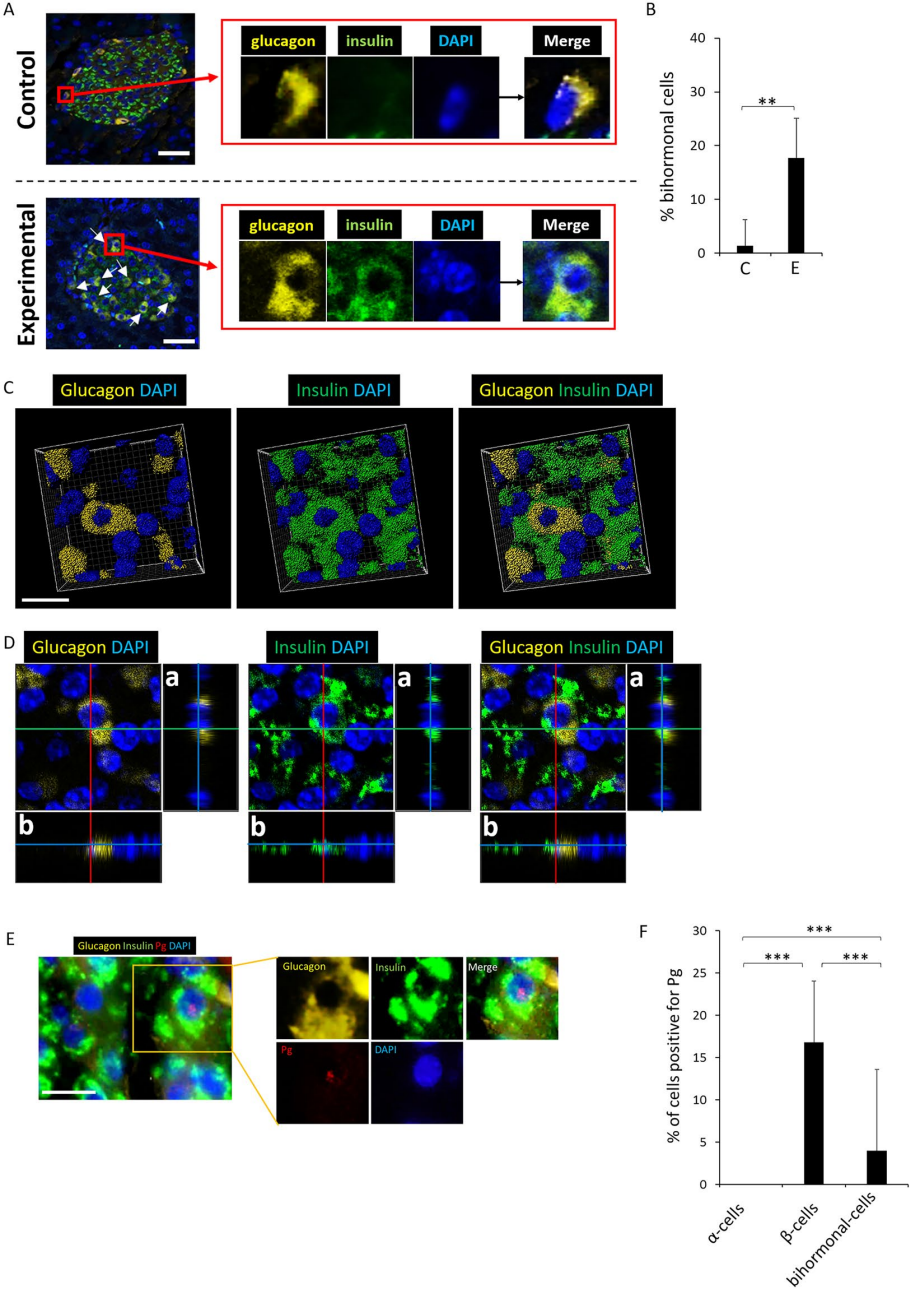
Bihormonal cells are evident in islets of the experimental mouse group. A low % of bihormonal cells contain Pg. (**A**) Upper panel is a representative islet from a control animal which does not exhibit bihormonal cells. Lower panel shows an islet from the experimental group. Emergence of bihormonal cells is evident (white arrows) in experimental animals as shown in a representative islets in bottom panel A. Yellow: glucagon, Green: insulin, Blue: nuclei. Representative of N = 7 animals/group. Scale bar represents 25μm. For separate channel images of 40×, see Fig. S2. (**B**) Percentage bihormonal cells in islets from control and experimental animals was calculated by identifying glucagon+/insulin+ cells divided by total number of glucagon+ cells per islet. 4–5 islets/animal. N = 7 animals/group. Y-axis: % bihormonal cells, X-axis: C represents control group and E represents experimental group. Data are presented as mean + S.D. **p < 0.01. (**C**,**D**) Representative 3-D image from confocal microscopy and orthogonal images respectively showing the presence of bihormonal cells in an islet from an experimental mouse. a & b are orthogonal projections showing the side views of the main image cut through along vertical (red) and horizontal (green) lines. (**E**) Pg presence is shown in a bihormonal cell of an experimental mouse. Yellow: glucagon, Green: insulin, Red: Pg, Blue: nucleus. Scale bar represents 10 μm. (**F**) Percentage of Pg positive α-, β- and bihormonal cells. Y-axis: % cells positive for Pg, X-axis: cell types. 449 α-cells, 968 β-cells and 72 bihormonal cells were counted. N = 5 experimental mice. Data are presented as mean + S.D. ***p < 0.001.

In the experimental group, 17.7% (S.D. ± 7.3%) of glucagon+ cells expressed both glucagon and insulin (Fig. 3A,B) whereas significantly fewer (mean 1.3%) glucagon+ cells expressed both insulin and glucagon in control group (p < 0.01) (Fig. 3B). These bihormonal cells were confirmed by 3-D and orthogonal analysis (Fig. 3C,D, respectively). A low percentage of bihormonal cells contain Pg/gingipain (Fig. 3E,F).

### Bihormonal cells express the β-cell transcription factor Nkx6.1

**Figure 4:**
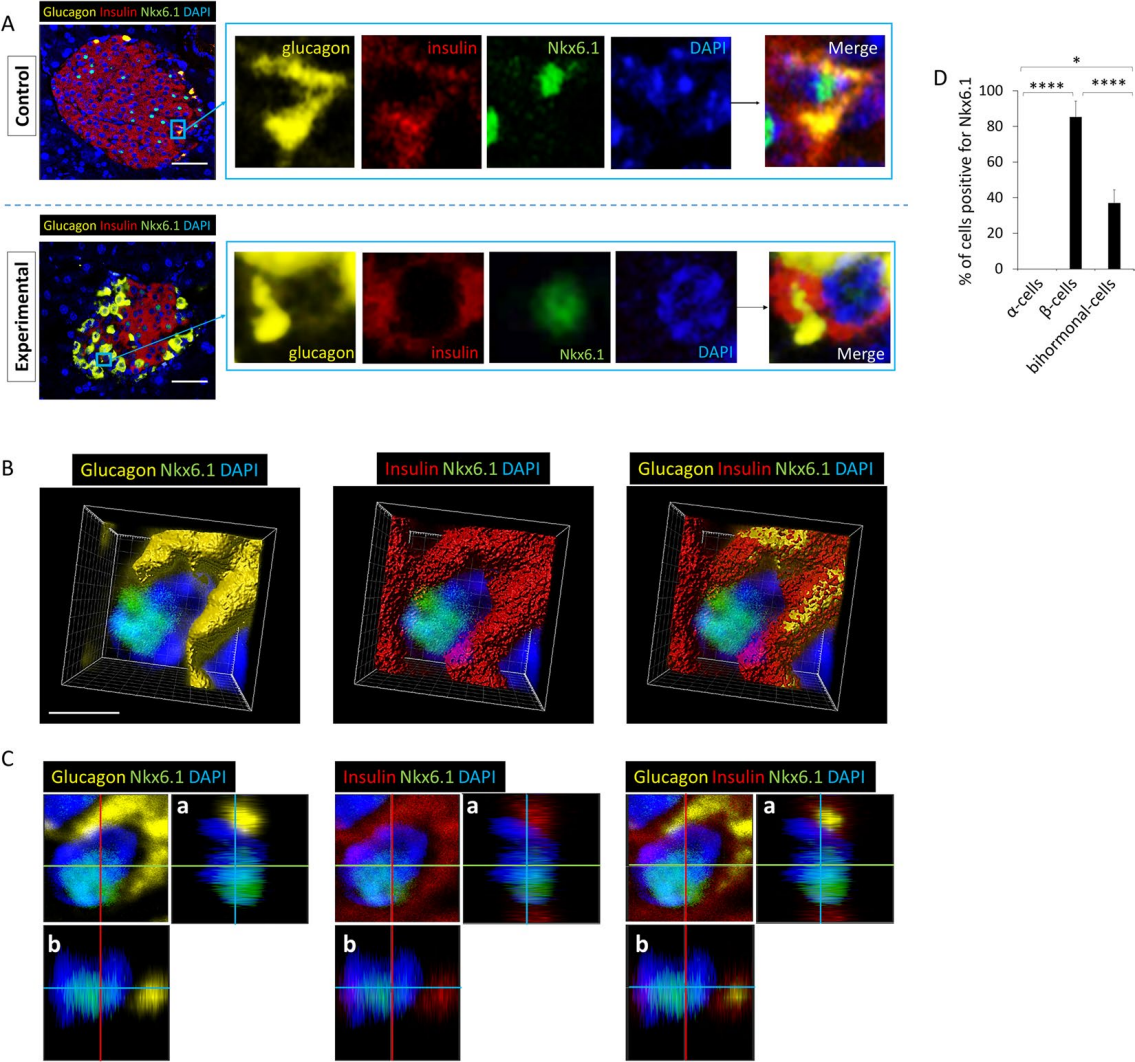
Bihormonal cells from both control and experimental groups express the β-cell transcription factor, Nkx6.1. (**A**) Upper panels are from control and lower panels are images from experimental groups. Nkx6.1 is expressed in bihormonal cells in experimental and control mice (N = 7). Scale bar represents 25μm. For separate channel images of 40×, see Fig. S3. (**B**) 3-D image from confocal microscopy of experimental mouse pancreatic islet cells stained for glucagon, insulin, Nkx6.1 and nuclei. Nkx6.1 is evident in the nucleus of a bihormonal cell. (**C**) Orthogonal analysis of a representative bihormonal cell expressing nuclear Nkx6.1. Each image corresponds to an image shown in (**B**). a & b are orthogonal projections showing the side views of the main image cut through along vertical (red) and horizontal (green) lines. Yellow: glucagon, Red: insulin, Green: Nkx6.1, Blue: nuclei. (**D**) % of cells positive for Nkx6.1. A total of 62 α-cells, 104 β-cells, and 15 bihormonal cells were counted. N = 4 experimental mice. Data show mean + S.D. *p < 0.05, ****p < 0.0001.

Nkx6.1 is a transcription factor that is expressed in islet β-cells to repress expression of the α-cell transcription factor, Arx, and is not expressed in mature α-cells. To confirm that bihormonal cells exhibit a characteristic of β-cells, we stained for expression of the β-cell specific transcription factor Nkx6.1. IF microscopy revealed that bihormonal cells that were positive for both insulin and glucagon co-expressed Nkx6.1 (Fig. 4A,D). 3-D and orthogonal analysis of bihormonal cells showed the intra-nuclear localization of Nkx6.1 (Fig. 4B,C respectively).

### Pg/gingipain is identified in human pancreata of both diabetic and non-diabetic subjects

We obtained a total of 45 postmortem human pancreata samples of which 32 were FFPE (19 Non-DM, 13 DM) and 13 frozen (11 Non-DM, 2 DM). Thus, of these 45 samples, 30 were from non-diabetic (Non-DM) and 15 from diabetic (DM) subjects (Table 1). In Non-DM subjects, 23% had Pg in the pancreas (Pg positive) and 77% were Pg negative (Table 1). In DM subjects, 40% were Pg positive and 60% Pg negative (Table 1). The Chi-square analysis reveals a Chi-square value of 5.662 with 1 degree of freedom. The two-tailed P value equals 0.01. Thus, the proportion of subjects with Pg was significantly higher in DM compared to Non-DM groups.

**Table 1:** Distribution of Pg+ and Pg− subjects grouped by Non-DM and DM status.

	Non-DM (N = 30)		DM (N = 15)		Total (N = 45)	
	Pg+	Pg-	Pg+	Pg−	Pg+	Pg−
# of subjects	7	23	6	9	13	32
% Pg+	23%	77%	40%	60%	29%	71%

Results from detection of Pg by IF microscopy (N = 32) and qPCR (N = 13). Chi-square analysis.

Overall Pg/gingipain was identified by immunofluorescence microscopy or by qPCR in 13 of 45 (29%) of human pancreata.

### Pg/gingipains are more strongly associated with β-cells than α-cells in human pancreata

**Figure 5:**
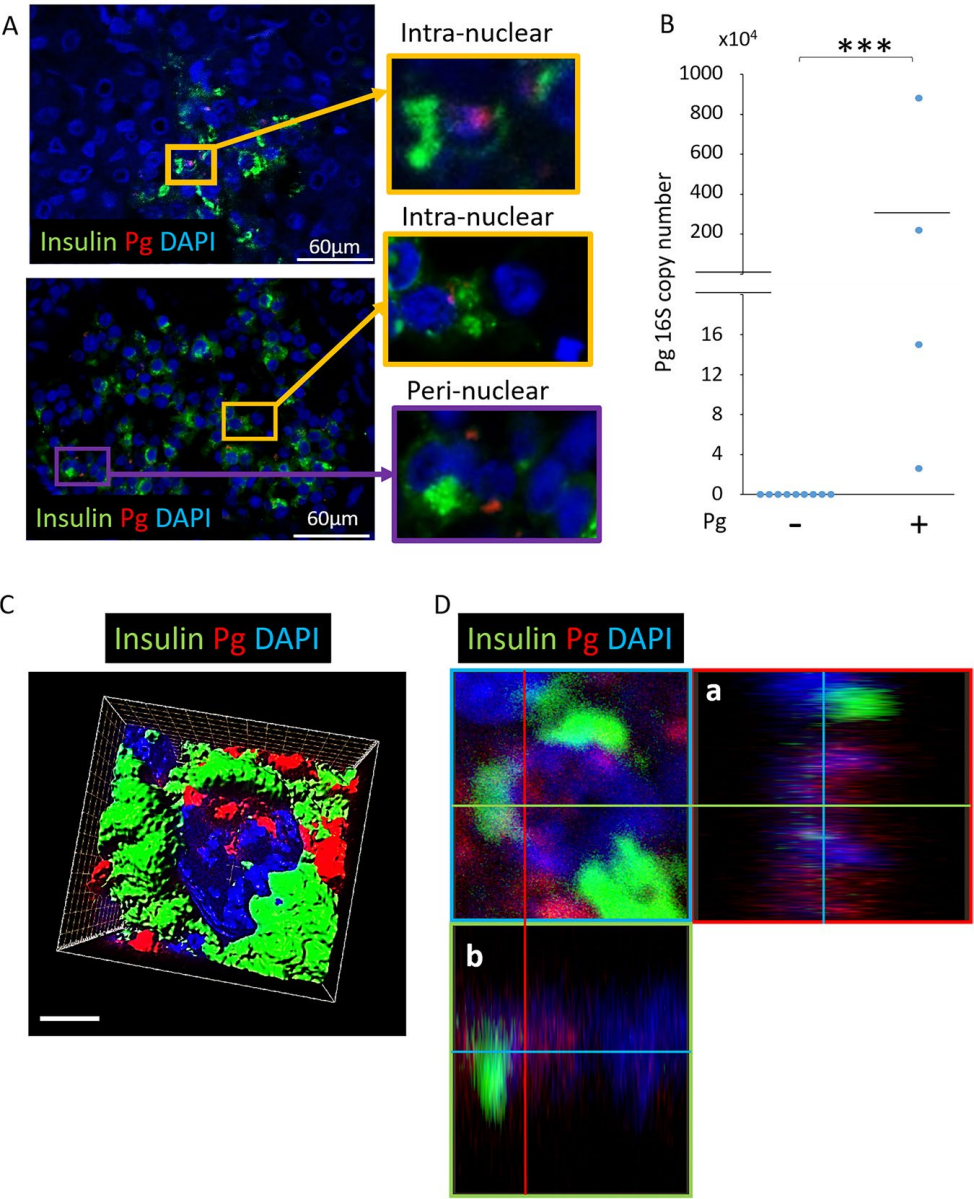
Human pancreatic islets are positive for Pg/gingipain. (**A**) 9 out of 32 FFPE samples from human subjects (age >50 y) show the presence of Pg/gingipain in islets. Representative IF images. Scale bar represents 60μm. (**B**) 4 out of 13 frozen samples were positive for Pg 16S rRNA genes detected by qPCR. Y-axis: copies/5 FFPE slides. ***p < 0.001. (**C**) 3-D image from confocal microscopy of human pancreatic islet composed from Z-sections, showing Pg/gingipain (red) associated with β-cells. Scale bar represents 5 μm. (**D**) Orthogonal analysis of a β-cell confirming intra-nuclear localization of Pg/gingipain. a & b represent orthogonal projections. Red: Pg, Green: insulin, Blue: nuclei.

2-D IF microscopy and 3-D confocal microscopy analysis of human pancreatic tissue sections show the presence of peri- and intra-nuclear Pg/gingipain in β-cells (Fig. 5A,C). As was observed in mouse islets, the presence of intra- and peri-nuclear localization was confirmed by orthogonal analysis (Fig. 5D). Of 13 frozen samples for which we performed qPCR analysis for Pg positivity, 4 were positive for Pg and 9 were negative. The copy number of the Pg 16S rRNA gene varied from 2.6 × 10^4^ to 8.8 × 10^6^ (mean 2.8 × 10^6^) (Fig. 5B).

**Figure 6:**
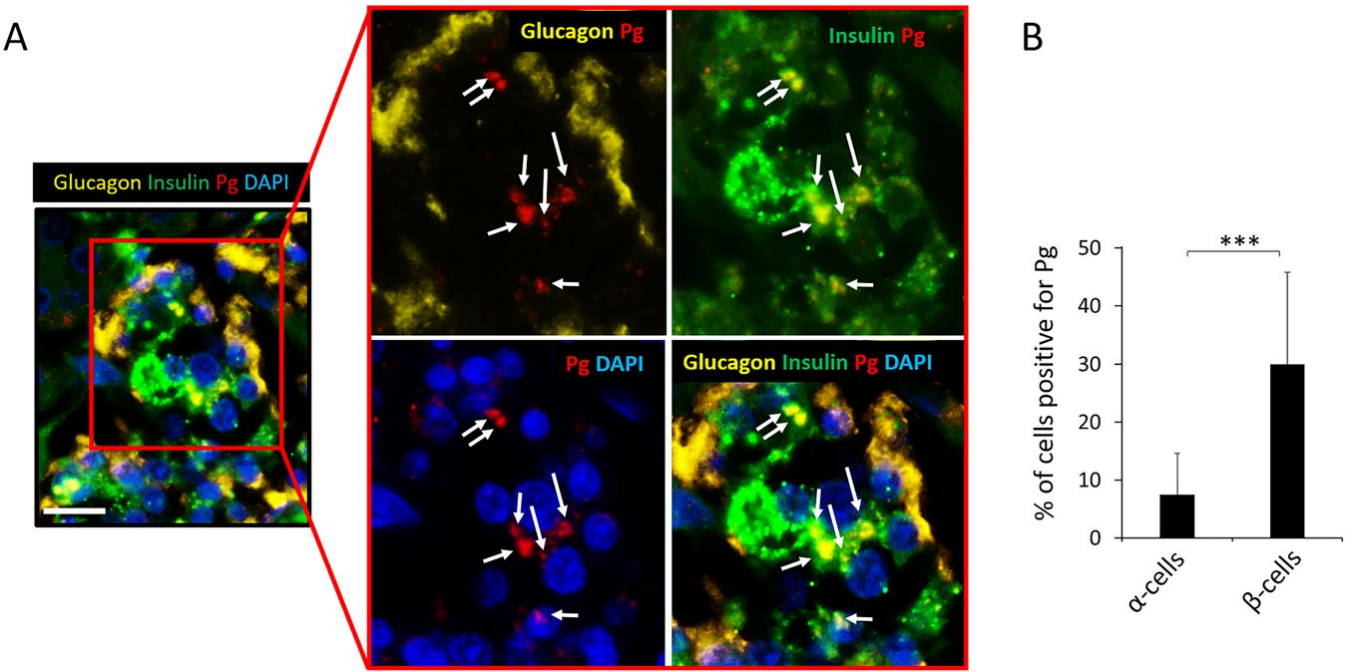
Pg/gingipain is localized more in β-cells compared with α-cells in human pancreata. (**A**) Representative IF image of human pancreata that are positive for Pg/gingipain. α-cells (yellow) do not have peri-nuclear or intra-nuclear Pg/gingipain but peri-nuclear Pg/gingipain is evident in β-cells (green). White arrows point to Pg. Yellow: glucagon, Green: insulin; Red: Pg, Blue: nuclei. Scale bar represents 25μm. (**B**) Percentage of α- and β-cells positive for Pg/gingipain. X-axis: α-cells and β-cells, Y-axis: percentage of α- or β-cells positive for Pg/gingipain. Cells were counted in 4 rectangular areas of 400μm X 300μm per sample in samples from 5 human subjects that were positive for Pg in islets. Total of 274 α-cells and 197 β-cells were counted. Data show mean ± S.D. ***p < 0.001.

We had determined that Pg/gingipain signal was exclusively localized to β-cells in samples from mice (Fig. 2) and addressed this same question in samples from humans. In contrast to what we found in mice, Pg/gingipain was mainly localized to β-cells but was also present in α-cells (Fig. 6A,B) in human islets. The mean percentage of Pg positive β- and α-cells was 30% and 7.5% respectively and this difference was statistically significant (p < 0.001) (Fig. 6B).

### Bihormonal cells are more abundant in pancreata of DM subjects

**Figure 7:**
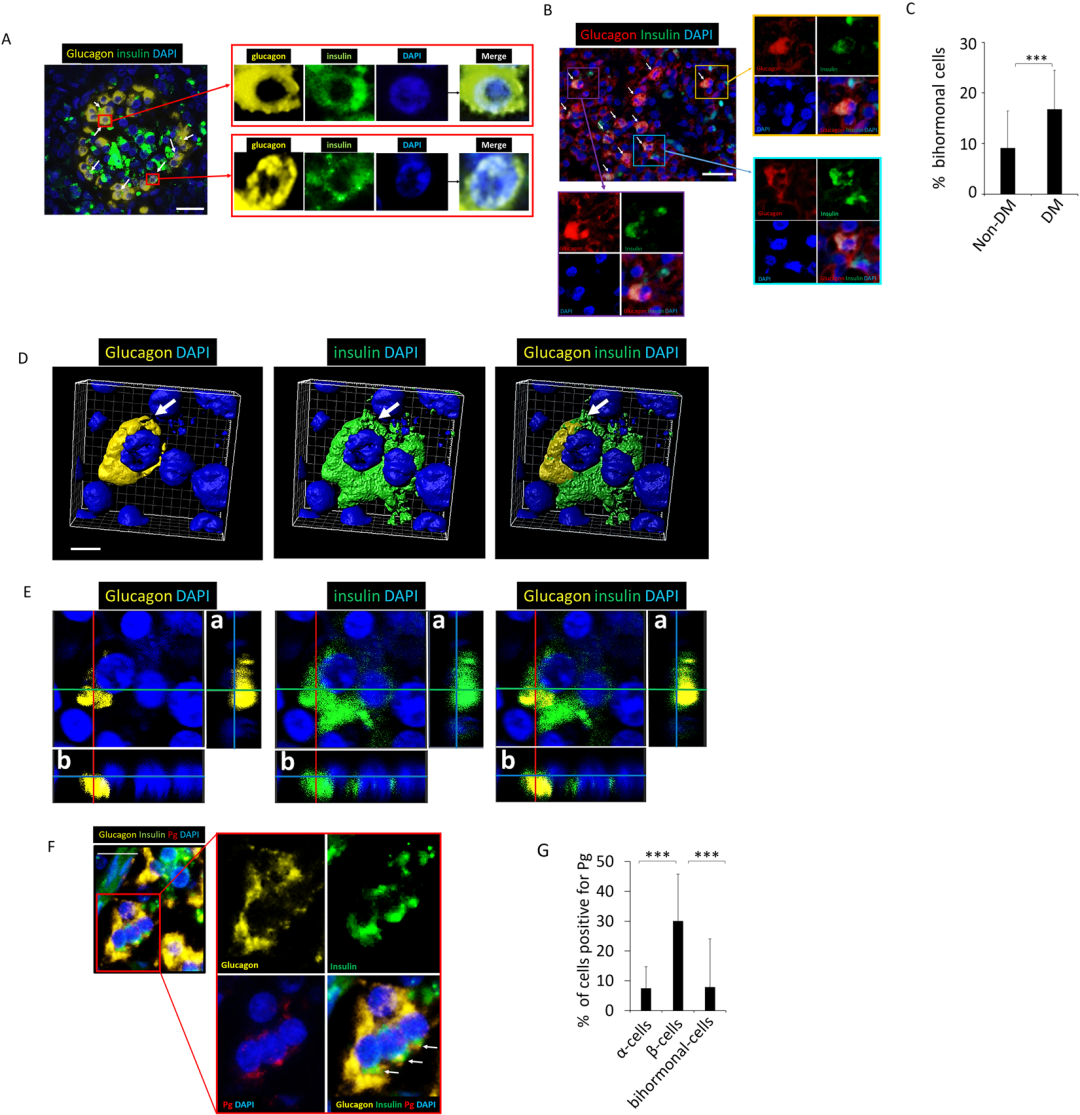
Bihormonal cells are detected in human pancreata from Non-DM and DM subjects. Pg is present in β-cells and in a low % of α-cells as well as bihormonal cells. (**A**) Representative result from IF microscopy showing the presence of bihormonal cells in human (Non-DM) post-mortem samples. White arrows indicate bihormonal cells. Scale bar represents 25 μm. Insets show glucagon+, insulin+, DAPI stained and merged images. Yellow: glucagon, Green: insulin, Blue: nuclei. Representative of N = 13 Non-DM samples. For separate channel images of 40×, see Fig. S4. (**B**) Bihormonal cells detected in pancreata of human DM subjects. White arrows point to bihormonal cells. Insets show glucagon+, insulin+, DAPI stained and merged images. Red: glucagon, Green: insulin, Blue: nuclei. Representative of N = 12. Scale bar represents 25 μm. (**C**) The percentage of bihormonal cells is significantly higher in DM compared with Non-DM subjects (***p < 0.001). % bihormonal cells was determined by counting the number of cells positive for insulin and glucagon divided by the number of cells positive for glucagon. A total of 429 and 469 α-cells were counted in Non-DM and DM subjects respectively. Two fields of 400 × 300 μm rectangle area were counted per subject. Data show means ± S.D. (**D**) 3-D images from confocal microscopy of glucagon+, insulin+ bihormonal cells (white arrows). Scale bar represents 5 μm. (**E**) Orthogonal analysis of bihormonal cell shown in (**D**). a & b represent orthogonal projections. Yellow: glucagon, Green: insulin, Blue: nuclei. (**F**) Representative image of bihormonal cells positive for Pg/gingipain. Yellow: glucagon, Green: insulin, Red: Pg/gingipain, Blue: nuclei. (**G**) Percentage of Pg positive α-, β- and bihormonal cells. Y-axis: % cells positive for Pg, X-axis: cell types. 347 α-cells, 444 β-cells and 51 bihormonal cells were counted. N = 5 DM subjects. Data are presented as mean + S.D. ***p < 0.001.

Bihormonal cells were detected in human post-mortem pancreata from Non-DM (Fig. 7A) and DM subjects (Fig. 7B). The percentage of bihormonal cells was significantly higher in DM (17 ± 7.6%) compared with Non-DM subjects (9 ± 7.2%) (p < 0.001) (Fig. 7C). 2-D IF images (Fig. 7A,B) as well as 3-D and orthogonal analysis (Fig. 7D,E, respectively) show the presence of bihormonal cells. IF microscopy data indicate that Pg/gingipain is present in β-cells and in a low percentage of α-cells as well as bihormonal cells (Fig. 7F,G).

### Bihormonal cells in human pancreata express the Nkx6.1 transcription factor

**Figure 8:**
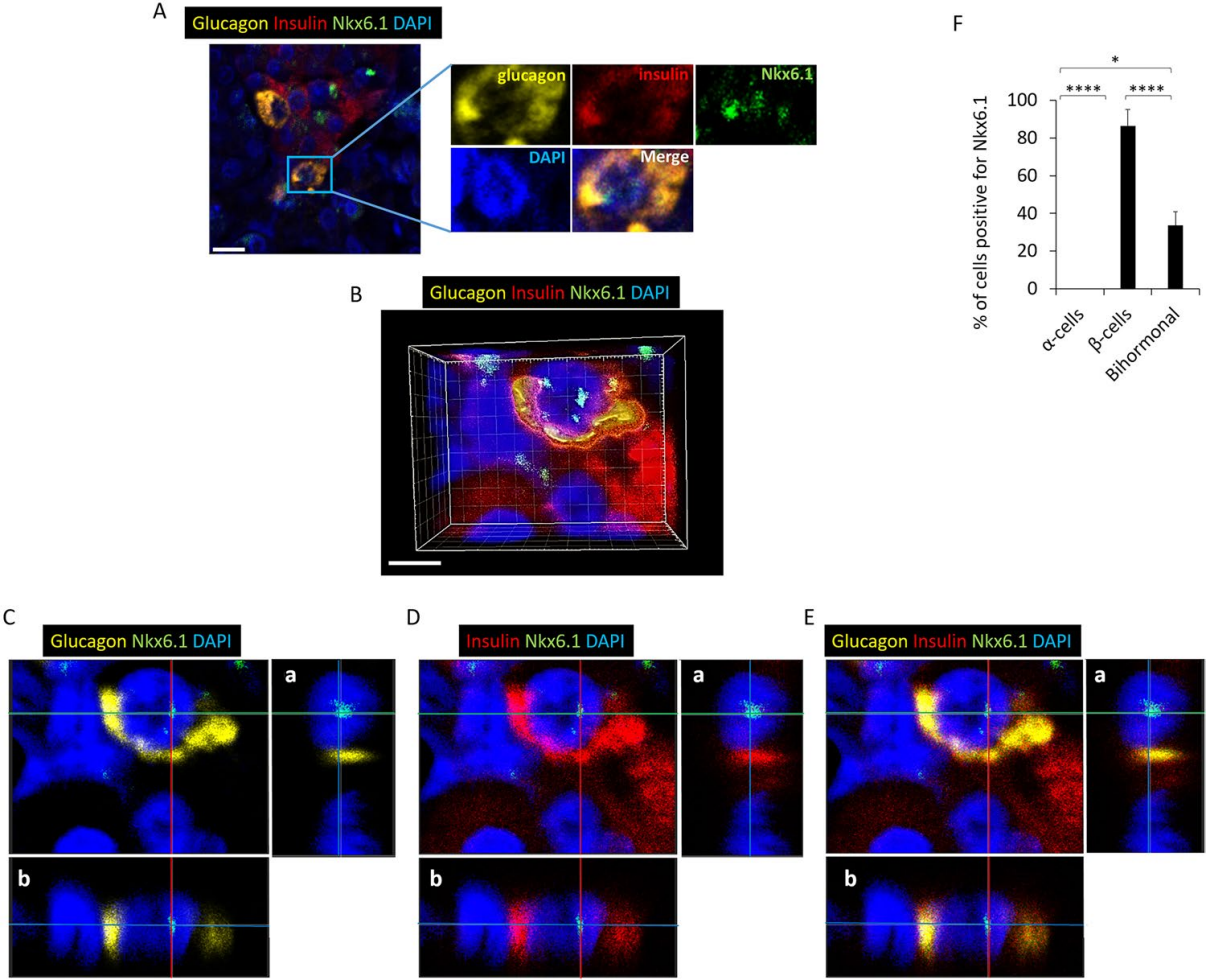
Human pancreatic bihormonal cells express Nkx6.1. (**A**) Representative images of bihormonal cells in DM subject expressing Nkx6.1 detected by IF microscopy. Scale bar represents 10 μm. For separate channel images of 40×, see Fig. S5. (**B**) 3-D image from confocal microscopy of a bihormonal cell expressing Nkx6.1. Scale bar represents 5 μm. (**C**) Orthogonal analysis of bihormonal cell staining for glucagon, Nkx6.1 and DAPI, (**D**) for insulin, Nkx6.1 and DAPI, and (**E**) for glucagon, insulin, Nkx6.1 and DAPI. a & b represent orthogonal projections. Yellow: glucagon, Red: insulin, Green: Nkx6.1, Blue: nuclei. (**F**) % of cells positive for Nkx6.1. A total of 76 α-, 118 β- and 30 bihormonal cells were counted. N = 4 DM subjects. Data show mean ± S.D. *p < 0.05, ****p < 0.0001.

2-D, 3-D and orthogonal analysis show β-cell transcription factor Nkx6.1 expression in the nucleus of bihormonal cells in human pancreata (Fig. 8A–E). The mean percentages of cells positive for Nkx6.1 were 0% for α-cells, 86% for β-cells and 34% for bihormonal cells (Fig. 8F).

### A strong correlation exists between Pg invasion and the emergence of bihormonal cells in both human and mice

**Figure 9:**
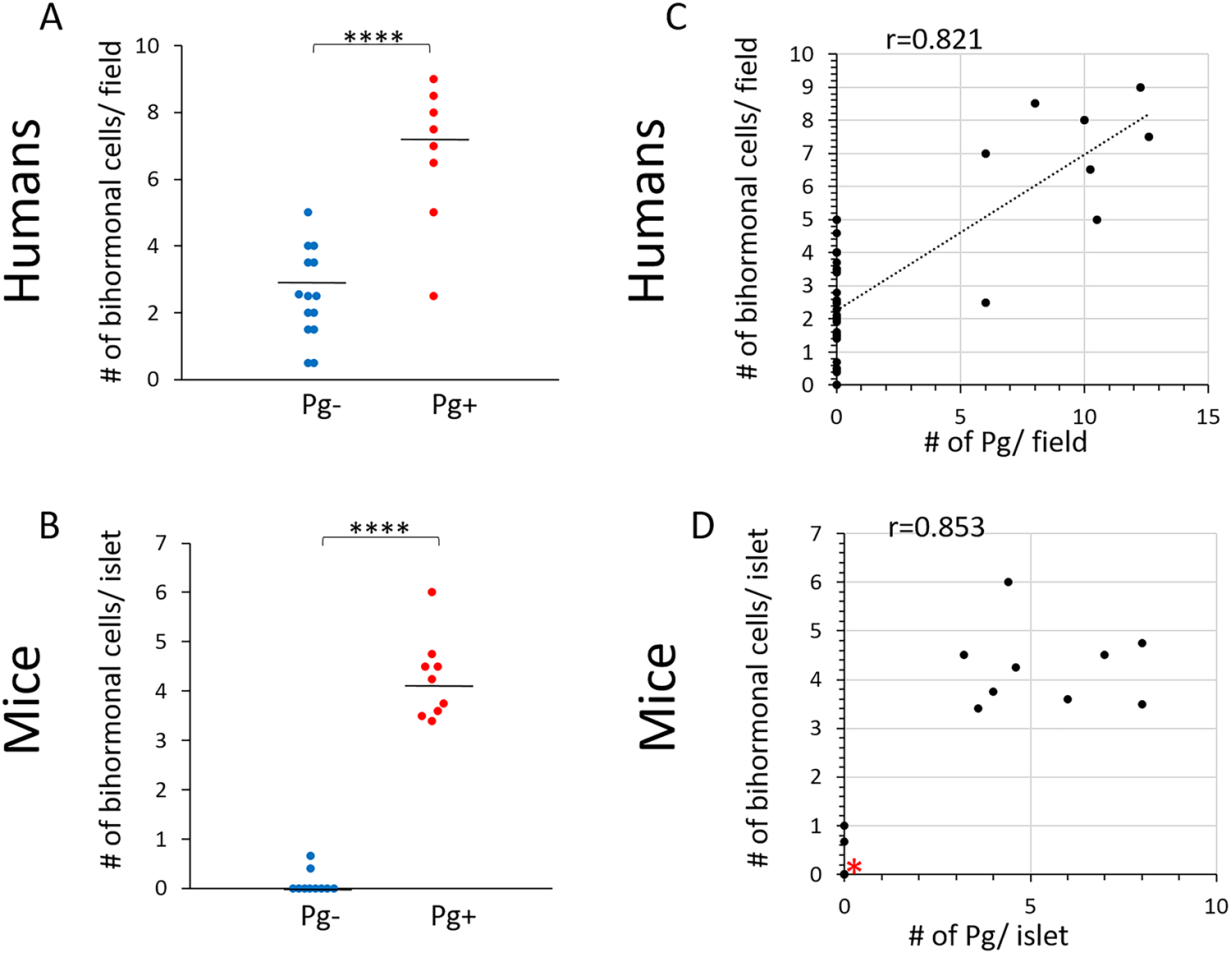
The number of bihormonal cells is significantly higher in pancreatic samples that contain Pg in both humans and mice. A strong correlation exists between the number of Pg/gingipain and emergence of bihormonal cells in both humans and mice. (**A**,**B**) Scatter plots showing the number of bihormonal cells/field (400 × 300 μm) in Pg− and Pg+ samples in human post-mortem samples and mice pancreatic islets. Total of 22 human samples and 19 samples from mice were examined. (N = 14 for Pg- and N = 8 for Pg+ for humans and N = 10 for Pg- and N = 9 for Pg+ for mice). Mann-Whitney test indicates ****p < 0.0001 for both humans and mice. (**C**) Pearson’s correlation analysis showing a strong correlation between the number of Pg and the number of bihormonal cells per field in humans (r = 0.821, ****p < 0.0001). (**D**) Spearman’s correlation analysis indicates a strong correlation between these two variables in mice (r = 0.853, ****p < 0.0001). 8 samples are overlapping at zero Pg and zero bihormonal cells (red asterisk).

We next determined the relationship between the number of bihormonal cells and the presence or absence of Pg in the pancreas in both humans and mice (Fig. 9A,B). In both humans and mice, the number of bihormonal cells was significantly higher in Pg+ compared with Pg- samples (p < 0.0001).

A correlation analysis was performed to determine if there is a relationship between the number of Pg and the number of bihormonal cells. This analysis showed a strong correlation between these two variables in both mice and human samples (correlation coefficient r = 0.821, p < 0.0001 for humans; r = 0.853, p < 0.0001 for mice) (Fig. 9C,D).

### Mice receiving oral application of Pg develop glucose intolerance, insulin resistance, hyperinsulinemia while maintaining normal glycemic levels

**Figure 10:**
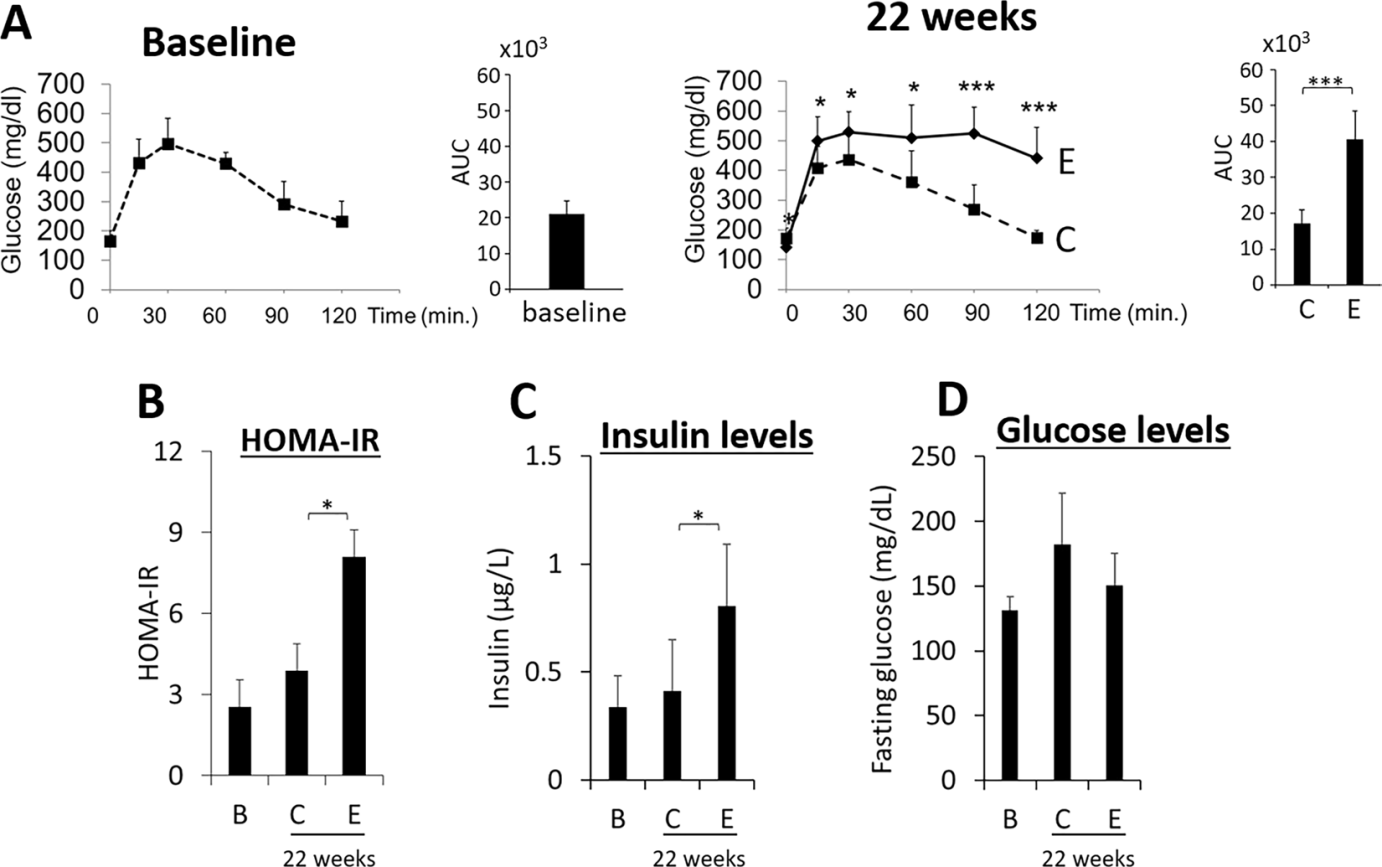
Animals with oral application of Pg develop severe glucose intolerance, IR and hyperinsulinemia but maintain normal glucose levels. (**A**) Animals with oral application of Pg (solid line, designated as **E**) develop glucose intolerance compared with control (dotted line, designated as **C**) by week 22 (N = 10 for each group). AUC stands for area under the curve. *p < 0.05, ***p < 0.001. (**B**) Insulin resistance (HOMA-IR). (**C**) Fasting insulin and (**D**) glucose levels were determined at week 1 (baseline B) and 22 weeks. Data are shown as mean ± S.D. in (**B**–**D**). *p < 0.05.

Oral application of Pg 3 times per week (Mondays, Wednesdays, Fridays) for 22 weeks in mice resulted in glucose intolerance (Fig. 10A), insulin resistance (Fig. 10B) and hyperinsulinemia (Fig. 10C) indicating increased β-cell release of insulin to maintain the normal glucose levels (Fig. 10D)^11^.

## Discussion

Nearly 50% of human adults in the U.S. have periodontitis^24^ with 20% having moderate, and 10% severe periodontitis. Periodontitis is a disease characterized by destruction of gingiva and tooth-supporting bone by a host immunological response to periodontal pathogens. It is reported that periodontal bacteria (gram-negative bacteria) are translocated to the liver^1^, aorta^2^, and arteries^3,4^ via the systemic circulation^25–27^ or possibly via the gut axis^1^, but the effects of periodontal pathogens in these tissues are poorly understood. We have recently reported the presence of Pg/gingipain in the brain (hippocampus) of young wild type mice that were administered Pg orally 3 times per week for 22 weeks and these mice developed neuropathology consistent with that of Alzheimer’s disease^5^. Our current study is the first to show the intra-cellular, peri-nuclear and intra-nuclear localization of a periodontal pathogen/product in pancreatic β-cells in mice as a result of oral application of Pg as well as in human pancreatic β-cells in both non-diabetic and diabetic subjects. Interestingly, the presence of Pg/gingipain in 29% of the randomly selected human pancreata used in this study corresponds to the percentage of humans with moderate to severe periodontitis. Unfortunately, in our current study, data regarding the periodontal condition were not available from these subjects. Nonetheless, given that the primary niche of Pg is the oral cavity, Pg/gingipain identified in the human pancreas most likely originated from this site.

In humans, the presence of Pg in the oral cavity or increased level of serum antibody to Pg is associated with pancreatic cancer^10^ but the actual presence of Pg in pancreatic cancer tissues has not been reported. However, a periodontal pathogen *Fusobacterium* sp. has been detected in pancreatic ductal adenocarcinomas and in pancreatic cystic neoplasm cyst fluid^8,9^. The result of our analysis of human post-mortem pancreatic samples is the first to show the presence of a periodontal pathogen in non-cancerous subjects, and specifically in human islets.

We used both a mouse monoclonal antibody (61BG1.3) which is specific to an epitope in the beta-adhesin domain of gingipains (G907-T931) and a rabbit polyclonal antibody raised against the active site of His sequence of gingipain^28^ to confirm the presence of Pg/gingpains in islets. Although positive signals using these antibodies reflect the presence of gingipains, this does not preclude the presence of whole bacteria residing peri-nuclearly or intra-nuclearly in β-cells. Gingipains are part of the outer cell membrane or reside inside membrane vesicles and thus it is possible that our staining detects whole Pg, a fragment of Pg cell membrane and/or secreted gingipains. Pg is a non-motile, coccobacillus gram-negative obligate anaerobe with an approximate size of 1.2 μm × 0.5 μm with an outer membrane vesicle of approximately 40 nm^29^. Since the size of a human β-cell is approximately 10–12 μm with a nuclear diameter of 6.0–8.0 μm^30–32^, our antibodies may detected whole Pg. In support of this, we detected Pg specific 16S rRNA genes in both mouse and human islets. However, we are not certain if this reflects the presence of either actively dividing or dormant bacteria.

Identification of a periodontal pathogen or its product in the nucleus of pancreatic islet cells is surprising. However, Pg is known to invade host cells such as epithelial^7^ and endothelial cells *in vitro*^33^. Further, a heterodimer derived from gingipain RgpA designated as HRgpA is known to enter the nucleus of epithelial cells and localize in and around the nucleus of these cells *in vitro*^7^. We recently reported the peri- and intra-nuclear localization of Pg/gingipain in neurons, microglial cells and astrocytes in the brain (hippocampus) of mice orally administered Pg^5^.

A number of bacteria and their products are known to enter and reside in host cell cytoplasm or the nucleus^34,35^. These bacteria include *E. coli*^36,37^, *Legionella penumophila*^38^ and its product OrfX which specifically targets the nucleus^39^, and *Rickttsia richettsii* which can invade the nucleus and form endonuclear colonies in human cells^40,41^. The mechanism that facilitates entry of bacteria or its product, referred to as “nuclear modulin (nucleomodulin),” into the nucleus is not clear as nucleomodulins usually lack a classical nuclear localization sequence (NLS)^42^. Pg/gingipains also do not have canonical NLS^7^. Study of the mechanism by which Pg and/or gingipain gains access to the nucleus primarily in β-cells may give us insight into the development of prediabetes and diabetes associated with oral bacteria. It is not clear why Pg/gingipain specifically invades β-cells in mice and significantly more in β- than α-cells in humans.

Bihormonal cells are considered an intermediate stage in α- to β-cell transdifferentiation^16^ and may be important as a β-cell compensatory response to IR^22,43^. Thus, the emergence of bihormonal cells is potentially a physiologically important mechanism to cope with IR and/or loss of functional β-cells. IF and confocal microscopy with orthogonal analysis showed nuclear localization of Nkx6.1 in a low percentage of bihormonal cells in both human and experimental mice. Nkx6.1 is a transcription factor specific to β-cells within the islets, which binds and inhibits transcription of the α-cell specific Arx gene thereby repressing α-cell identity^44^. However, secretion of insulin from bihormonal cells isolated from mice subjected to oral application of Pg has yet to be confirmed by functional analysis.

The literature reporting quantitative data on human bihormonal cells is limited^21,22^, and previous studies have not considered the possibility that bacterial pathogens may contribute to their emergence. Two possible means to induce conversion of α-cell to β-cell or β-cell to α-cell have been identified: (1) near complete ablation of β-cells using chemical agents^13,45,46^ and (2) forced expression/deletion of β- or α-cell specific transcription factors and/or deletion of Dnmt1 or FOXO1 transcription factor in β-cells in mice^14–16,18,19^. We found that Pg/gingipain is not detected in mouse α-cells and at a low % in human α-cells, making it unlikely that Pg directly induces α- to β-cell transdifferentiation. However, whether the emergence of bihormonal cells is based on α- to β-cell transdifferentiation or β-cell de-differentiation/conversion in the context of an oral bacteria reaching the pancreas will require additional studies as some of our bihormonal cells in mice and human did contain Pg/gingipain. Thus, the study using lineage tracing methods would be necessary to identify the direction or bi-direction of islet cell transdifferentiation and/or dedifferentiation. In our mouse model of experimental periodontitis the percentage of β-cell destruction/apoptosis was a mean of 8%^12^ and yet bihormonal cells (mean 17.7% of total count of glucagon+ cells) emerged, suggesting that near complete destruction of β-cells may not be necessary for the emergence of bihormonal cells in our model system. Interestingly, Pg-LPS has been shown to impact gene expression by significantly reducing DNA methyltransferase (DNMT1) expression^47^. Inactivation of both Dnmt1 and Arx in adult mice has been shown to drive transdifferentiation/conversion of α- to β-cells^16^. It has also been shown that β-cells deficient in DNMT1 are converted to α-cells^19^. Thus, Pg with cell membrane associated LPS present in the nucleus of β-cells may be altering the methylation signature and thus β- and α-cell identity. Further, intra-cellular localization of Pg/gingipain suggests other epigenetic functions of Pg/product. For example, it is reported that Pg decreases trimethylation of histone H3K4^48^. Furthermore, treatment of cultured pancreatic islets with a histone methyltransferase inhibitor results in colocalization of both glucagon and insulin in mouse islets, i.e. bihormonal cells^49^. Based on these data, it is possible that Pg translocation from the oral cavity to the nuclei of islet cells may lead to the decreased trimethylation of H3K4 and thereby contribute to the emergence or maintenance of bihormonal cells. In summary, we present data obtained by IF, confocal microscopy and qPCR that Pg is present in human pancreata and is at a higher incidence in the pancreata of DM than Non-DM humans.

This is the first study to investigate the presence and intra-cellular localization of a periodontal pathogen and/or its product in human pancreata and also in the pancreas of an animal model of experimental periodontitis. Our data indicate that Pg/gingipain is translocated from the oral cavity to pancreatic islets and localized primarily in β-cells in both humans and mice. In this process, emergence of bihormonal cells occurs. The results from a recent study show that Pg expresses dipeptidyl peptidase 4 (DPP4) which degrades incretins and thus reduces insulin levels which is accompanied by a substantially elevated glucose levels at a 15 min time point following oral glucose administration^50^. PgDPP4 does not appear to account for our results since our experimental mice show high insulin levels and normal glycemic levels, thus suggesting that non-DPP4 factors/effects may be involved. However, we can’t exclude the possibility that PgDPP4 may be present but not contributing to net effects on insulin levels. Finally, based on the published literature on nuclear invasion of bacteria/products in various cell types, it is interesting to speculate that Pg/gingipains may epigenetically influence development of bihormonal cells.

## Methods

### Animals

This study was carried out in strict accordance with the recommendations outlined in the Guide for the Care and Use of Laboratory Animals of the National Institutes of Health. The protocol was approved by the Institutional Animal Care and Use Committee at the University of Illinois at Chicago (Protocol approval #15–142). Twenty 8-week old male C57BL/6 mice were used for this study. Oral application of Pg was performed 3 times per week for 22 weeks. At baseline and at week 22, a glucose tolerance test was performed by intraperitoneal injection of 50% dextrose solution (2 g/kg body weight) and glucose levels in tail blood were determined at 15, 30, 60, 90 and 120 min post-dextrose injection using a glucometer. Fasting insulin levels were determined by ELISA (10-1247-01, Mercodia AB, Uppsala, Sweden) and insulin resistance was calculated using the homeostasis model assessment (HOMA-IR)^51^.

### Human pancreata

A total of 45 human post-mortem pancreata samples (age >50) were obtained from the Department of Pathology at the University of Illinois at Chicago. Of these 45 samples, 32 were paraffin embedded and 13 were from frozen tissue samples. The identity of subjects were de-identified and only the age, the diagnosis or absence of diabetes, types of diabetes and cause of death were provided. Exclusion criterion was subjects with diagnosis of pancreatic or any other cancer. The use of human post-mortem pancreatic tissues was approved by the Institutional Review Board at the University of Illinois at Chicago (Protocol approval # 2018-0759). Appropriate informed consent was obtained from next of kin for autopsies and all methods were carried out in accordance with University of Illinois Hospital Institutional guidelines and regulations involving dead bodies.

### Bacterial culture and oral application of Pg

Briefly, Pg (strain W83) was grown anaerobically as described in our previous study^11^. Cell density was determined by spectrophotometry at an optical density of 550 nm based on a standard curve established using colony forming units (CFU). 1 × 10^9^ Pg were transferred to microfuge tubes, vortexed briefly and centrifuged at 10,000 g for 2 min at 4 °C. Supernatants were discarded and the pellets were re-suspended in 4 ^o^C PBS, and then re-pelleted by centrifugation. Supernatants were removed, and bacteria re-suspended in 100 μl of 2% carboxymethyl cellulose (CMC) in PBS and tubes immediately placed on ice until administered to mice. 100 μl of Pg in CMC containing 10^9^ Pg (experimental group) or CMC alone (vehicle alone control group) was placed (2 applications of 50 μl) in the oral cavity every other day (Monday, Wednesday, and Friday) for 22 weeks. Animals were sacrificed using isoflurane anesthesia followed by cervical dislocation following 22 weeks of Pg application and pancreata were removed and fixed in paraformaldehyde to be embedded in paraffin.

### Mouse pancreata

Mouse pancreata samples were obtained from C57BL/6 WT mice that received Pg orally (experimental animals, N = 10) or vehicle alone (control animals N = 10)^11^. Pancreata from these animals were harvested, fixed in formalin and embedded in paraffin (FFPE).

### Immunofluorescence, confocal microscopy, and orthogonal analysis of human and mouse pancreas

Immunofluorescence microscopy (IF) was performed to detect Pg/gingipain and α- and β-cells. Briefly, 5μm thick sections were first de-paraffinized with xylene and rehydrated through a series of decreasing percentages of ethanol. Antigen retrieval was performed by microwaving sections in 1 mM ethylenediaminetetraacetic acid (EDTA), pH 8.0 or in 10 mM citrate buffer pH 6.0 for 5 min repeated 4 times. Cell and nuclear membrane permeabilization was performed by incubating sections in 0.25% Tween 20 in PBS for 30 min. To detect Pg/gingipain, tissue sections were incubated overnight at 4 ^o^C either with mouse anti-Pg monoclonal antibody (DSHB, Iowa City, Iowa) or rabbit anti-Pg polyclonal antibody as primary antibody^28^ at a dilution of 1:100, followed by donkey anti-mouse conjugated to Alexa Fluor 647 (A31571, Invitrogen, Grand Island, NY) or donkey anti-rabbit IgG conjugated to Alexa Fluor 594 (A21206, Invitrogen, Grand Island, NY) respectively as secondary antibody at room temperature for 1 hr. To detect β-cells, tissue sections were incubated overnight at 4 °C with goat anti-insulin antibody (sc-7838, Santa Cruz Biotechnology, Inc., Dallas, TX) at a concentration of 1:100, followed by donkey anti-goat IgG conjugated to Alexa Fluor 488 (A11055, Invitrogen, Grand Island, NY), as secondary antibody at room temperature for 1 hr. To detect α-cells, tissue sections were incubated overnight at 4 ^o^C with anti-glucagon antibody at a concentration of 1:100 (ABCAM, Cambridge, MA), followed by donkey anti-mouse IgG conjugated to Alexa Fluor647 (A31571, Invitrogen, Grand Island, NY) as secondary antibody. To detect the β-cell specific transcription factor Nkx6.1, goat anti-Nkx6.1 antibody (AF5857, R&D Systems, Inc, Minneapolis, MN USA) was used as primary antibody at a concentration of 1:100. Sections were incubated for 1.5 hr in a humidified chamber at room temperature, followed by anti-goat IgG conjugated with Alexa Fluor 488 at a 1:800 dilution as secondary antibody. Isotypic controls were used to determine non-specific binding of antibodies for all experiments. Nuclei were identified using DAPI as described^12^. For 3-D image reconstruction, an isovoxel data-set was acquired. Orthogonal analysis were performed based on compiled z-sections (0.08μm step size) obtained by confocal microscopy using the Imaris software (Bitplane, Inc) version 7.7.2.

For samples from mice, the percentage of cells that exhibited perinuclear/intranuclear Pg/gingipain in α- and β-cells was calculated under 40X magnification by counting the number of insulin+/Pg+ cells divided by the total number of insulin+ cells per islet or glucagon+/Pg+ cells divided by the total number of glucagon+ cells. A total of 5 islets per mouse were examined in 5 mice/group.

In human post-mortem pancreatic samples, the percentage of α- and β-cells exhibiting Pg+ was calculated as for mouse samples except that cells were counted in four rectangular areas of 400 × 300 μm per sample.

The percentage of bihormonal cells in mice were counted under 40X magnification by identifying glucagon+/insulin+ cells relative to the total number of glucagon+ cells per islet. 4–5 islets/animal were examined in 7 animals/group.

For human samples, the percentage of bihormonal cells positive for Pg/gingipain was determined by counting the number of cells identified by immunofluoresnce microscopy that were positive for both insulin and glucagon staining relative to the number of cells that stained positive for glucagon in a 400 × 300 μm rectangle under 40 X magnification.

### Detection of Pg specific 16S rRNA genes by qPCR

Genomic DNA was extracted from formalin fixed paraffin embedded (FFPE) mouse pancreatic samples using a MaxwellRSC device and the Maxwell DNA FFPE kit (AS1450, Promega Corporation, Madison, WI) and from human frozen pancreatic samples following homogenization in TE Buffer pH 8.0 using an MP FatsPrep-24 5 G homogenizer for 30 seconds. Homogenized samples were extracted using the Maxwell 16 Tissue DNA Purification Kit based on manufacturer’s instructions (Promega Corporation, Madison, WI). Primers and probes were synthesized as described by Boutaga *et al*.^52^. The sequences of the Pg16S rRNA gene forward primers were GCGCTCAACGTTCAGCC, reverse primers were CACGAATTCCGCCTGC, and probe was CACTGAACTCAAGCCCGGCAGTTTCAA. PCR primers and TaqMan probe for detection of Pg were custom made by Integrated DNA Technologies (IDT, Coralville, IA) with a 6-FAM fluorescent label and both Zen internal and 3’ Iowa Black fluorescence quenchers. A synthetic double-stranded DNA standard for 16S rRNA gene was synthesized as a gBLOCK fragment (IDT, San Jose, CA)^5^. The standard contained 243 bp of the16S rRNA gene from the Pg strain W83, and greater than 60 bp of adjacent DNA on either side of the target sites. The double-stranded gBLOCK oligonucleotide was diluted across 8 orders of magnitude, and used as a standard for quantitation of Pg 16S rRNA genes in genomic DNA extracts. The concentration of the standards was assessed using Qubit fluorimetry, and the copies per microliter assessed by estimation of the DNA weight/molecule based on sequence and sequence length and the DNA concentration. Together with a dilution series, absolute quantification (copies per microliter of nucleic acid extract) of samples was assessed through linear regression analysis from the standard curve. All assays were performed in technical triplicates. PCR amplification was performed in a total reaction mixture volume of 25 μl. The reaction mixtures contained 12.5 μl of 2x TaqMan universal PCR master mix (4304437, Thermo Fisher Scientific), 300 nM each Pg-specific primer, 100 nM Pg-specific probe, and purified DNA. The samples were subjected to an initial amplification cycle of 50 °C for 2 min and 95 °C for 10 min, followed by 40 cycles at 95 °C for 15 s and 60 °C for 1 min^5^.

### Statistical analysis

Nonparametric data, Pg 16S copy number in mice and human samples as well as ipGTT, glucose, insulin and HOMA-IR, were evaluated by the Mann-Whitney U-test for two-group comparisons. The proportion of subjects with Pg+ in pancreas was analyzed between DM and Non-DM groups using a Chi-square test. Shapiro-Wilk test was used to determine the distribution of the data. Based on the results of Shapiro-Wilk test, the Pearson’s correlation analysis was used to analyze the correlation between the number of bihormonal cells and the number of Pg in humans, and Spearman’s correlation analysis was used for mice samples. All other statistical analyses were done using a Student’s t-test with a cutoff value of p < 0.05 considered significant.

## Supplementary information

Supplementary information.

## Data Availability

The data sets analyzed for this study are available from the corresponding author upon reasonable request.
