# Supplementary material for: Identification of a periodontal pathogen and bihormonal cells in pancreatic islets of humans and a mouse model of periodontitis
Source: Sci Rep. 2020 Jun 19;10:9976. doi: 10.1038/s41598-020-65828-x (PMC7305306; doi:10.1038/s41598-020-65828-x)

Supplementary information

Identification of a periodontal pathogen and bihormonal cells in pancreatic islets of humans and a mouse model of periodontitis

Vladimir Ilievski[1], Peter T. Toth[2,3], Klara Valyi-Nagy[4], Tibor Valyi-Nagy[4], Stefan J. Green[5,6], Rosann S. Marattil[7], Haider W. Aljewari[8], Barton Wicksteed[9], Neil M. O'Brien-Simpson[10], Eric C. Reynolds[10], Brian T. Layden[9], Terry G. Unterman[9,11], Keiko Watanabe[1*]

[1]Department of Periodontics, College of Dentistry, University of Illinois at Chicago, Chicago, Illinois, United States of America

[2]Department of Pharmacology, College of Medicine, University of Illinois at Chicago, Chicago, Illinois, United States of America

[3]Fluorescence Imaging Core Facility, University of Illinois at Chicago, Chicago, Illinois, United States of America

[4]Department of Pathology, College of Medicine, University of Illinois at Chicago, Chicago, Illinois, United States of America

[5]Department of Biological Sciences, University of Illinois at Chicago, Chicago, Illinois, Unites States of America

[6]DNA Core Facility, University of Illinois at Chicago, Chicago, Illinois, United States of America

[7]Undergraduate Program, University of Illinois at Chicago, Chicago, Illinois, United States of America

[8]Post-Gradulate Program in Periodontics, College of Dentistry, University of Illinois at Chicago, Chicago, Illinois, United States of America

[9]Division of Endocrinology, Diabetes & Metabolism, College of Medicine, University of Illinois at Chicago, Chicago, Illinois, United States of America

[10] Melbourne Dental School, University of Melbourne, Melbourne, Victoria, Australia.

[11]Jesse Brown VA Medical Center, Chicago, Illinois, United States of America

Supplementary Figure S1. Pg is localized in β-cells. (Separate channel images for Fig. 2A)

Representative immunofluorescence microscopy images showing the presence of Pg in β-cells in experimental mice. Yellow: glucagon, Green: insulin, Red: Pg, Blue: nuclei. Scale bar represents 50μm. Square represents an example of β-cells with Pg.

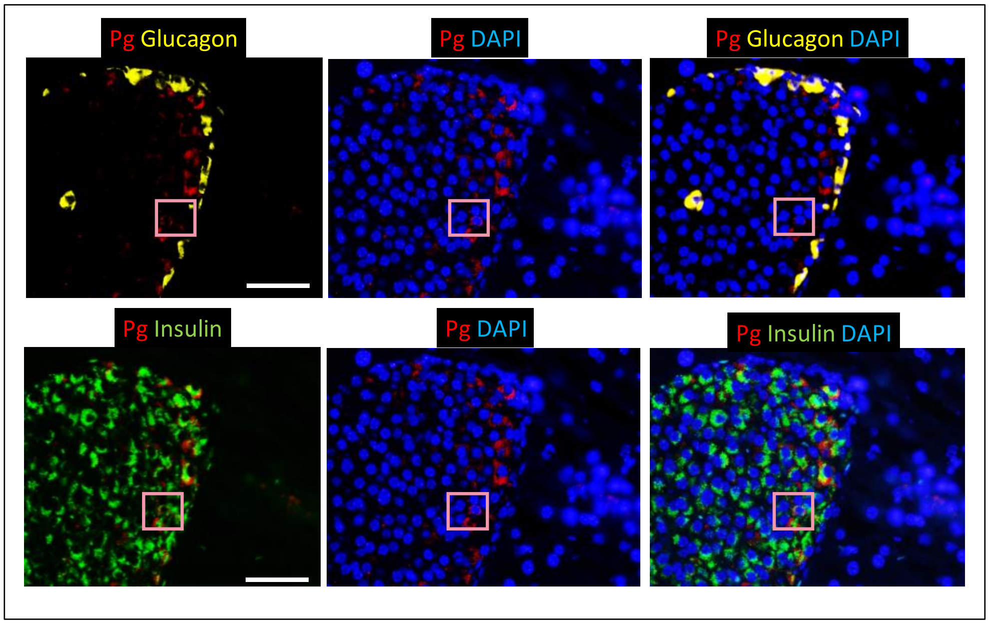

Supplementary Figure S2. Bihormonal cells are evident in islets of the experimental group. (Separate channel images for Fig. 3A)

Representative immunofluorescence microscopy images from a control animal (upper panel) which does not exhibit bihormonal cells and experimental animal (lower panel) which exhibit bihormonal cells. Yellow: glucagon, Green: insulin, Blue: nuclei. Scale bar represents 25μm. Rectangle represents an example of a bihormonal cell containing Pg (lower panel).

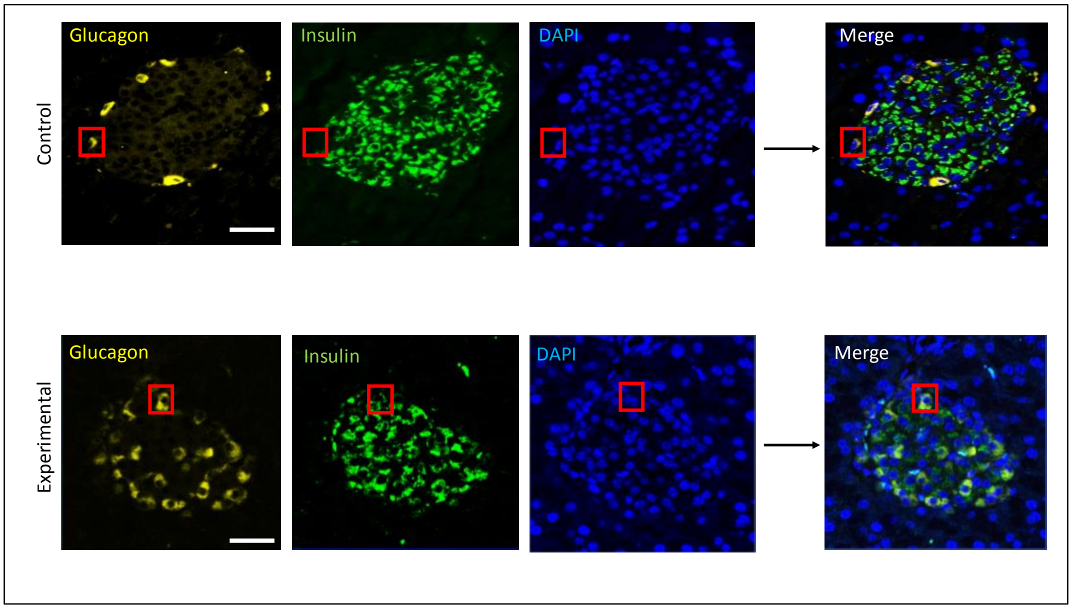

Supplementary Figure S3. Bihormonal cells from both control and experimental groups express the β-cell transcription factor, Nkx6.1. (Separate channel images for Fig 4A)

Mouse bihormonal cells express Nkx6.1. Representative immunofluorescence microscopy images showing the presence of Nkx6.1 in β-cells and bihormonal cells. Yellow: glucagon, Red: insulin, Green: Nkx6.1, Blue: nuclei. White rectangles show bihormonal cells expressing Nkx6.1. Scale bar represents 25μm.

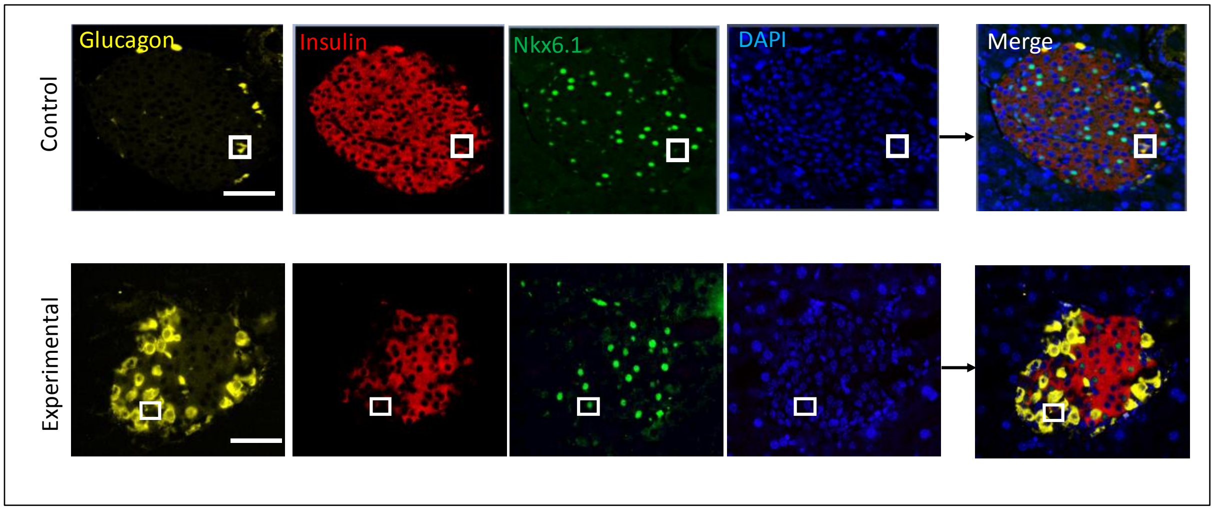

Supplementary Figure S4. Bihormonal cells are present in human pancreata from Non-DM subjects. (Separate channel images for Fig. 7A)

Representative immunofluorescence microscopy images showing the presence of bihormonal cells. Yellow: glucagon, Green: insulin, Blue: nuclei. Red squares show examples of bihormonal cells. Scale bar represents 25μm.

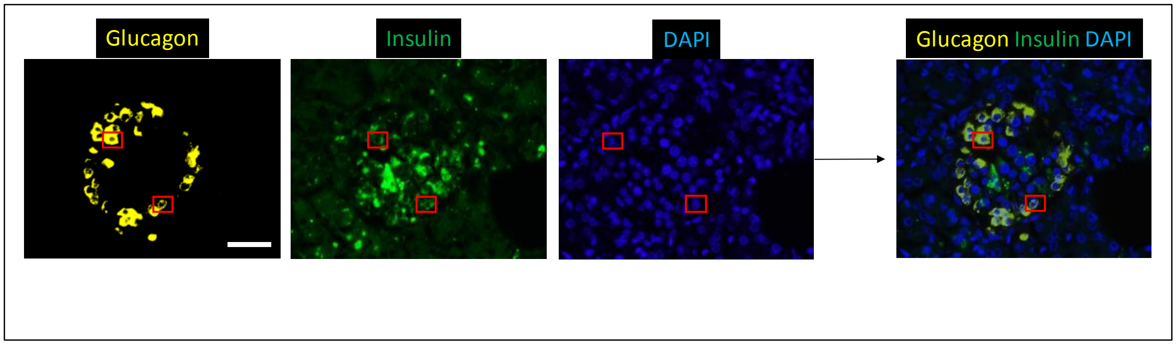

Supplementary Figure S5. Human pancreatic bihormonal cells express Nkx6.1.
(Separate channel images for Fig. 8)

Representative images of bihormonal cells in human DM subjects expressing Nkx6.1. Yellow: glucagon, Red: insulin, green: Nkx6.1, Blue: nuclei. Rectangle shows an example of a bihormonal cell positive for Nkx6.1. Scale bar represents 10µm.

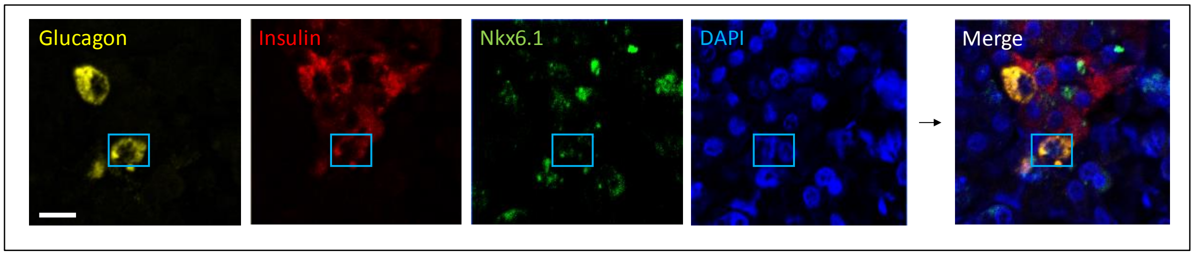

Supplement: Supplementary file 1 — Supplementary information. [file 41598_2020_65828_MOESM1_ESM.pdf]
